# Spatiotemporal regulation of multipotency during prostate development

**DOI:** 10.1242/dev.180224

**Published:** 2019-10-15

**Authors:** Elisavet Tika, Marielle Ousset, Anne Dannau, Cédric Blanpain

**Affiliations:** 1 Laboratory of Stem Cells and Cancer, Université Libre de Bruxelles (ULB), Brussels 1070, Belgium; 2 WELBIO, Université Libre de Bruxelles, Brussels 1070, Belgium

**Keywords:** Stem cells, Prostate development, Basal multipotency, Cell fate, Lineage tracing, Mouse

## Abstract

The prostate is formed by a branched glandular epithelium composed of basal cells (BCs) and luminal cells (LCs). Multipotent and unipotent stem cells (SCs) mediate the initial steps of prostate development whereas BCs and LCs are self-sustained in adult mice by unipotent lineage-restricted SCs. The spatiotemporal regulation of SC fate and the switch from multipotency to unipotency remain poorly characterised. Here, by combining lineage tracing, whole-tissue imaging, clonal analysis and proliferation kinetics, we uncover the cellular dynamics that orchestrate prostate postnatal development in mouse. We found that at an early stage of development multipotent basal SCs are located throughout the epithelium and are progressively restricted at the distal tip of the ducts, where, together with their progeny, they establish the different branches and the final structure of prostate. In contrast, pubertal development is mediated by unipotent lineage-restricted SCs. Our results uncover the spatiotemporal regulation of the switch from multipotency to unipotency during prostate development.

## Abstract (precis)

**Highlighted Article:** A combination of lineage tracing and whole-mount imaging uncovers how the multipotency of basal stem cells is regulated during postnatal prostate development in mouse.

## INTRODUCTION

The prostate is a glandular organ of the male reproductive system in mammals. It resides around the urethra and underneath the bladder (Abate-Shen and Shen, 2000; Marker et al., 2003). Its main function is to produce approximately one-third of the seminal fluid. By supplying essential nutrients, enzymes and ions, prostatic secretions ensure the survival of spermatozoa until reproduction.

The adult prostate epithelium in rodents and humans comprises three distinct lineages: basal cells (BCs), luminal cells (LCs) and rare neuroendocrine cells (Abate-Shen and Shen, 2000). BCs form a discontinuous layer of cells, characterised by the expression of K5 (Krt5), K14 (Krt14) and p63 (Trp63), surrounding the LC layer (Kasper, 2008; Shen and Abate-Shen, 2010). LCs express keratin K8 (Krt8), K18 (Krt18), androgen receptor (AR) and secretory proteins such as prostate specific antigen (PSA) (Abate-Shen and Shen, 2000; Vashchenko and Abrahamsson, 2005). A few cells expressing both basal and luminal markers simultaneously, called intermediate cells, are presented in the developing and adult prostate in mice and humans (Garraway et al., 2003; Hudson et al., 2001; Taylor et al., 2010).

The mouse prostate is organised as bilaterally differing lobes, including the ventral prostate (VP), the dorsolateral prostate (DLP) and the anterior prostate (AP) (Abate-Shen and Shen, 2000; Marker et al., 2003). The epithelium forms glands that are organised in ductal-branched structures. The VP is found below the bladder on top of the urethra, whereas the DLP is extended on each side of the urethra and the AP is located along the seminal vesicles (Hayward et al., 1996). The prostate derives from epithelial budding of the endodermal urogenital sinus during embryonic development (Cunha et al., 1987; Timms et al., 1995; Timms, 2008). The urogenital sinus is committed to form prostatic epithelium on embryonic day (E)16 and the first epithelial buds can be observed at E17.5 (Timms et al., 1994). These buds grow into the mesenchyme generating the different lobes. The main ducts of each lobe are visible during embryonic development but they start to branch extensively after birth (Marker et al., 2003). By 8-10 weeks postnatally the mouse prostate development is entirely complete (Sugimura et al., 1986).

Lineage-tracing experiments have shown that the cellular turnover in adult prostate during homeostasis and regeneration is mediated by distinct populations of unipotent basal and luminal restricted stem cells (SCs) (Choi et al., 2012). In addition, rare bipotent basal and luminal SCs were also observed when lineage tracing was conducted with K5^+^ BCs or Nkx3-1^+^ LCs in adult mice during cycles of prostate regeneration following androgen administration (Lu et al., 2013; Wang et al., 2013). Genetic lineage-tracing experiments performed at an early stage of postnatal development [postnatal day (P)1] using K5 and K14 as well as K8 and K18-CreER to target the basal and luminal lineage, respectively, have been used to decipher the cellular and lineage hierarchy that controls prostate postnatal development (Ousset et al., 2012). This study revealed that BCs were heterogeneous during prostate postnatal development and contained basal multipotent SCs that differentiate into BCs and LCs, as well as unipotent basal and luminal progenitors. Mathematical modelling suggested that the apparent cellular heterogeneity of basal progenitors could be explained by the stochastic cell fate decision of a single multipotent progenitor (Ousset et al., 2012). The lack of information regarding the clone location along the ductal trees as well as the lineage tracing at a single time point (P1) prevented definitive conclusions from being drawn regarding the spatiotemporal regulation of cell fate during prostate postnatal development.

In this study, we combined different lineage-tracing strategies and developed whole-mount (WM) imaging techniques to analyse the heterogeneity of basal and luminal SCs and monitor their fate across the entire prostate epithelium at different time points during prostate postnatal development. This approach allowed us to assess whether SC fate, such as multipotency and unipotency, is regulated spatially and temporally and to determine when and where basal progenitors become lineage restricted during prostate postnatal development. We found that the proliferation and the fate of basal SCs change rapidly in a spatially and temporally regulated manner ensuring the harmonious postnatal growth of the prostate.

## RESULTS

### Morphometric analysis of prostate postnatal development

**Fig. 1.**
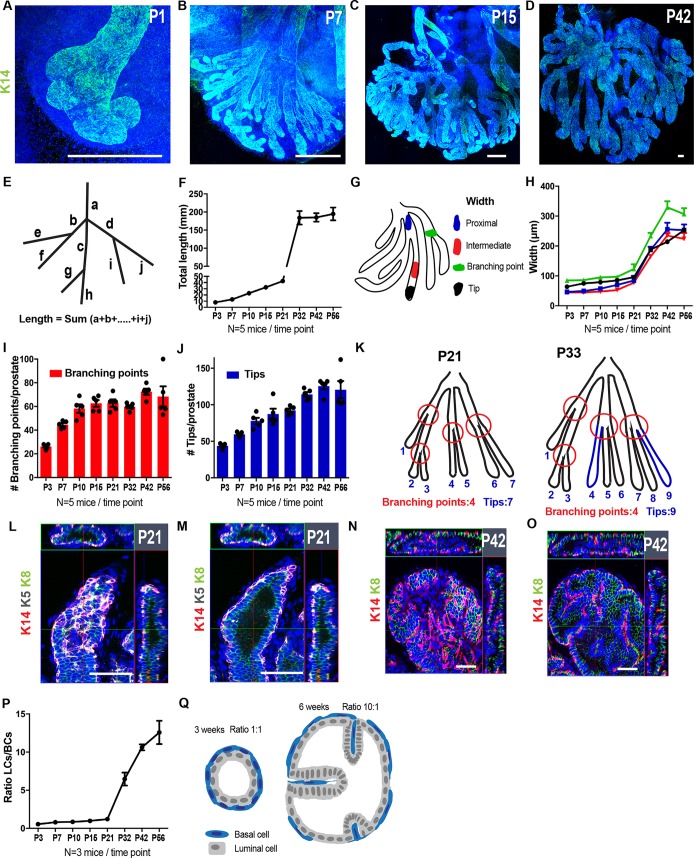
**Three-dimensional analysis of prostate postnatal development.** (A-D) Three-dimensional confocal microscopy analysis of WM of the VP at different time points during postnatal development. Tissue is stained with K14 (green) and Hoechst 33342 (blue). (E) Schematic of the ductal tree showing the method used to measure the length of the prostate epithelium as shown in F. (F) Measurement of the total length of the ductal tree at different times. (G) Schematic of the different ductal regions, where width was measured as shown in H. (H) Measurement of the width of the different regions of the ductal tree at different times. Five different measurements were taken per region, per sample. Line colours correspond to the key shown in G. (I) Total number of branching points per VP at different time points. (J) Total number of tips per VP at different times. (K) Schematic of the progression in the number of branching points and tips at P21 and P33. (L-O) Confocal images at P21 (L,M) and at P42 (N,O) at the upper layer (L,N) and the middle layer (M,O) of the ductal epithelium stained with K14 (red), K5 (grey; in L,M), K8 (green) and Hoechst 33342 (blue), showing the stretching of the basal compartment and progressive cavitation of the luminal compartment. (P) Ratio of LCs to BCs in the prostate epithelium at different time points. In total, 13,761, 23,447, 20,706, 30,756, 59,386, 48,658, 54,101 and 63,199 cells were counted for each time point (left to right). (Q) Scheme illustrating a cross-section of a prostate duct at P21 (left) and P42 (right). Data show mean±s.d. (F,I,J) or mean±s.e.m. (H,P). Scale bars: 200 μm (A-D); 50 μm (L-O).

The extensive postnatal growth of the murine prostate is mediated by SC activity that converts the embryonic prostate rudiment into a functional mature branched ductal network (Toivanen and Shen, 2017). How this postnatal SC activity is regulated remains currently poorly understood. In mice, neonatal development starts at P1 and continues until P20, followed by weaning (P21-P28) and puberty (P28-P42); males reach adult size and sexual maturity at P42-P60. To gain better insight into the epithelial architecture and the tubular network organisation of the developing prostate, we established a microdissecting protocol that allows isolation, staining, mounting and imaging of intact prostate lobes at different time points during postnatal development (Fig. 1A-D). We focused our analysis on the VP because its postnatal development begins immediately after birth (Timms et al., 1994). At P1, the VP had already undergone one to three bifurcations/branches and budding of new branches was clearly visible (Fig. 1A). The main branching events occurred during the first 15 postnatal days; after P15 the number of ducts did not further increase and the ducts grew only in size until reaching their final adult structure around 6-8 weeks postnatally (Fig. 1B-D). To understand the dynamics of tissue growth, we quantified several parameters that define precisely the overall tissue expansion. In particular, we measured the total length of all the ducts within the VP and the width of the epithelium in distinct regions at different times during postnatal prostate development (Fig. 1E,G). We observed linear tissue elongation until the onset of puberty, which usually takes place after weaning from P21 to P28 (Fig. 1F). The rate of growth increased between P21 and P42, which corresponds to the end of puberty, and then it decelerated until the length of the ductal tree reached a plateau (Fig. 1F). We measured the width of the ducts in the proximal, intermediate and distal parts as well as at the branching points (Fig. 1G). In all cases, we found that the width did not expand much until P21 and then increased from P21 to P42 to reach a plateau (Fig. 1H). To capture the evolution of the branching complexity of the ductal tree, we scored the total number of branching points and tips per prostate. We found that the number of branching points increased during the first 2 weeks of development whereas after P15 the total number of branching points was not further enhanced (Fig. 1I). The total number of tips continued to increase until the beginning of pubertal development (Fig. 1J) showing that new buds can emerge from the existing branching points (Fig. 1K). Confocal analysis of the tubular structures revealed that until P21 the tubes were composed of a single layer of LCs expressing K8 covered by a continuous layer of BCs expressing K5/K14 (Fig. 1L,M). However, from P21 to P42, growth was accompanied by a folding of the epithelium into the inner part of the tubes, increasing the area of LCs per duct and giving the appearance of tubular cavitation (Fig. 1N,O). This feature was observed along the whole length of the ducts. The increase in the width of the ducts was accompanied by stretching and elongation of BCs, leading to the formation of a discontinuous BC layer (Fig. 1N,O). By scoring the total number of BCs and LCs per imaging field, we observed that the ratio of LCs/BCs was about 1:1 until puberty and then increased to 6:1 at P33 and 10:1 at P42-P56 (Fig. 1P,Q). This morphological change coincides with the onset of puberty, suggesting that the hormonal alterations associated with this period induce a new mode of tissue morphogenesis that maximises the number of LCs per duct and the secretory capacity of the organ.

Altogether, these morphometric data show that the global architecture of the future adult prostate is established during the first part of postnatal development until the beginning of puberty, whereas the greatest tissue expansion and growth occur during pubertal development until adulthood.

### Spatiotemporal regulation of cell division during postnatal development

**Fig. 2.**
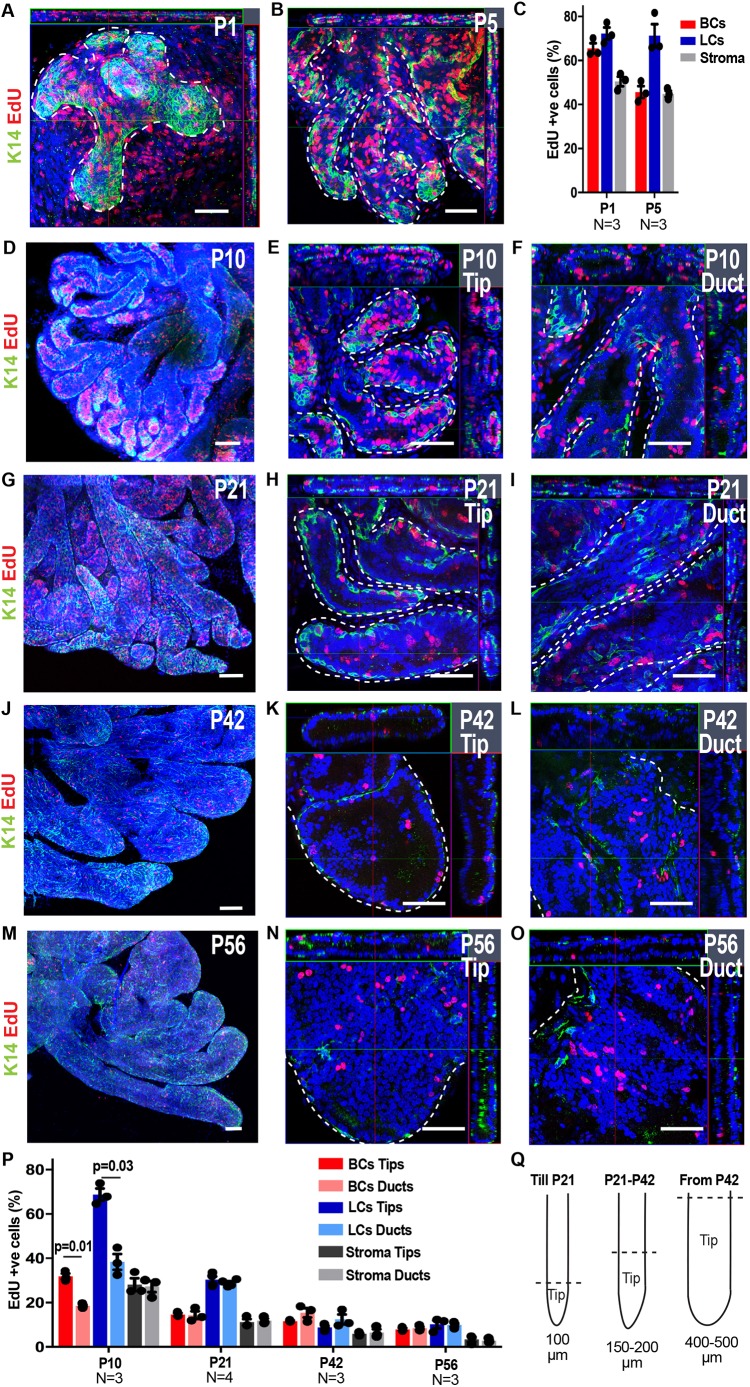
**Spatiotemporal regulation of cell proliferation during prostate postnatal development.** (A,B) Confocal images of EdU incorporation in the VP at P1 (A) and P5 (B). (C) Quantification of proportion of EdU-positive cells at P1 and P5 12 h following EdU administration. Mean±s.e.m. are shown from 6772 and 8928 cells counted from 3 mice for P1 and P5, respectively. (D-O) Confocal images of the proliferating cells at low magnification (D,G,J,M), at the tip (E,H,K,N) and at the duct (F,I,L,O) of the VP at different points during development. (P) Quantification of EdU-positive cells in tips and ducts 24 h following EdU administration at different time points during postnatal development. Mean±s.e.m. are shown from 31,916, 30,388, 27,759 and 21,029 cells counted at the different time points indicated (left to right). (Q) Schematic showing the distance from the distal tip of the ducts that is defined as the tip region for data analysis at different time points during development. In all image panels, K14 is visualised in green, EdU in red and nuclei were counterstained with Hoechst 33342 in blue. Dashed lines outline the ducts. *P*-values were calculated by Mann–Whitney test. Scale bars: 50 μm (A,B,E,F,H,I,K,L,N,O); 100 μm (D,G,J,M).

To unravel the proliferation kinetics and whether cell proliferation is coupled with tissue growth during prostate postnatal development, we administrated 5-ethynyl-2′-deoxyuridine (EdU) and evaluated its incorporation after a 12- or 24-h chase by WM immunostaining. At P1, proliferation was equally elevated in BCs and LCs, with more than 60% EdU-positive cells 12 h following EdU administration (Fig. 2A,C). At P5, cell proliferation continued to be intense, although BCs were proliferating less frequently than LCs (Fig. 2B,C). Stromal cells presented high levels of proliferation at these two time points as well (Fig. 2C). The difference in proliferation between BCs and LCs was observed throughout the remaining postnatal growth. Around P10, there was a higher proliferation rate at the tip region (Fig. 2Q) compared with the ductal region in both BCs and LCs, demonstrating that tissue proliferation is not stochastic all along the epithelium and is spatially regulated (Fig. 2D-F,P). The proliferation rate of stromal cells was similar at the tips and the ducts suggesting that the differences between epithelial cells were not the consequence of a higher proliferation of stromal cells at the tip region (Fig. 2P). From the onset of puberty (P21) until adulthood (P56), our analysis revealed that BC, LC and stromal cell proliferation decreased over time within the different regions until adulthood, when both BCs and LCs proliferated at a lower rate irrespective of tissue localisation (Fig. 2G-Q).

We then considered whether the increase of the LC/BC ratio observed during development could be a result of differential cell death between BCs and LCs. We found that overall tissue apoptosis was very low for both the basal and the luminal compartment, with a higher cell death rate in LCs, showing that the increase of the LC/BC ratio is not a result of preferential BC death (Fig. S1A-D).

### Spatiotemporal regulation of BC multipotency during prostate development

**Fig. 3.**
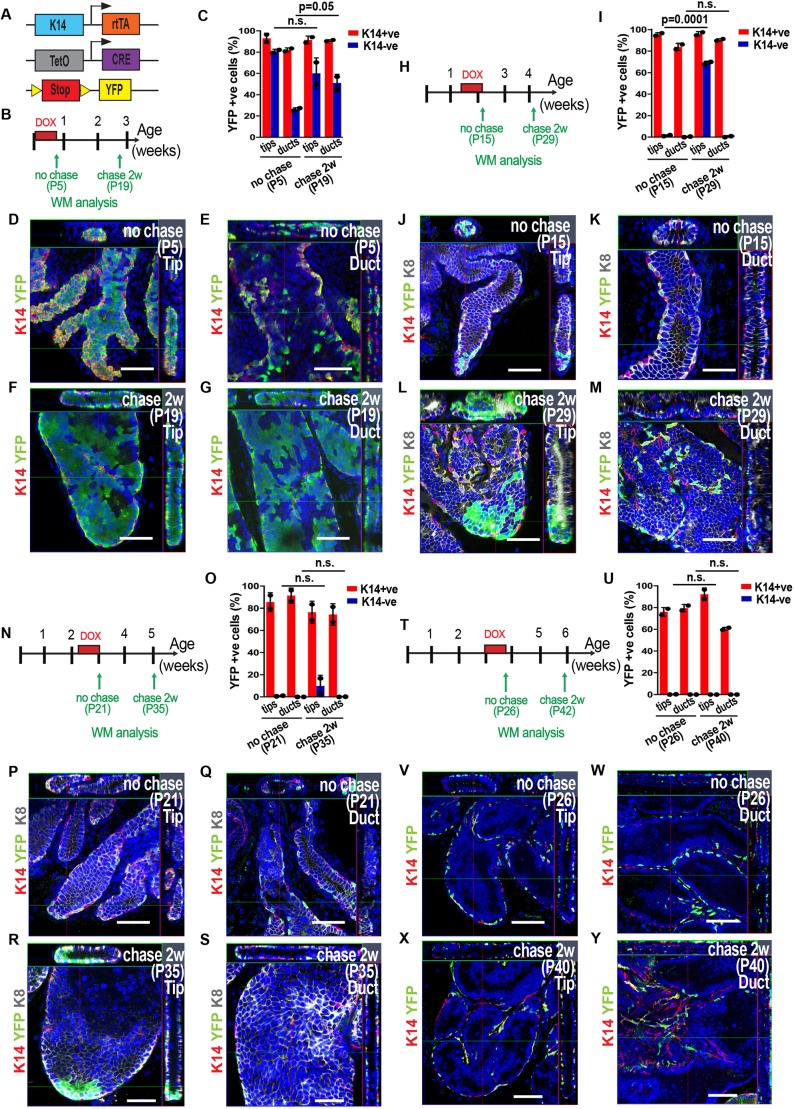
**Basal lineage tracing at saturation shows the spatiotemporal regulation of multipotency during prostate development.** (A) Genetic strategy used to induce YFP expression in most K14-expressing BCs at different time points during prostate development. (B) Protocol used to analyse saturation lineage tracing at early stage of postnatal development (P1-P5). (C) Quantification of YFP^+^ cells in BCs (K14^+^) and LCs (K14^−^) at the end of DOX administration (P5) and after 2 weeks of chase (P19) at the tip or the duct. In total, 19,322 (P5) and 23,298 (P19) cells were counted from 2 mice. (D-G) Confocal images of the VP at the end of DOX administration (P5; D,E) and after 2 weeks of chase (P19; F,G). (H) Protocol used to analyse saturation lineage tracing during mid postnatal development (P10-P15). (I) Quantification of YFP^+^ cells in BCs (K14^+^) and LCs (K14^−^) at the end of DOX administration (P15) and after 2 weeks of chase (P29) at the tip or the duct. In total, 23,362 (P15) and 24,712 (P29) cells were counted from 2 mice. (J-M) Confocal images of the VP at the end of treatment (P15; J,K) and after 2 weeks of chase (P29; L,M). (N) Protocol used to analyse saturation lineage tracing performed just before puberty (P16-P21). (O) Quantification of YFP^+^ cells in BCs (K14^+^) and LCs (K14^−^) at the end of DOX administration (P21) and after 2 weeks of chase (P35) at the tip or the duct. In total, 44,114 (P21) and 36,544 (P35) cells were counted from 2 mice. (P-S) Confocal images of the VP at the end of DOX administration (P21; P,Q) and after 2 weeks of chase (P35; R,S). (T) Protocol used to analyse saturation lineage tracing performed at the onset of puberty (P21-P26). (U) Quantification of YFP^+^ cells in BCs (K14^+^) and LCs (K14^−^) at the end of DOX administration (P26) and after 2 weeks of chase (P40) at the tip or the duct. In total, 51,064 (P26) and 44,781 (P42) cells were counted from 2 mice. (V-Y) Confocal images of the VP at the end of Dox administration (P26; V,W) and after 2 weeks of chase (P40; X,Y). In all image panels, K14 is visualised in red, YFP in green, K8 in grey (in J-M,P-S) and nuclei were counterstained with Hoechst 33342 in blue. Data show mean±s.e.m. *P*-values were calculated by Mann–Whitney test. n.s., not significant. Scale bars: 50 μm.

To assess the fate and multipotency of BCs during postnatal prostate development at the organ level, we performed doxycycline (DOX)-inducible lineage tracing of BCs using K14rtTA/TeO-Cre/Rosa-YFP mice at different time points of postnatal development and YFP expression was monitored by WM confocal analysis (Fig. 3A,B,H,N,T). This genetic strategy resulted in a high rate of BC recombination (at least 80% of YFP^+^ BCs) and the ability to define BC fate behaviour in relation to their relative position along the ductal tree. Administration of DOX at P1 and WM analysis 1 day later showed that only BCs were YFP labelled, demonstrating the specificity of labelling using this approach (Fig. S1E,F). When we labelled BCs from P1 to P5 and examined the samples after DOX treatment (P5) and after 2 weeks of chase (P19), we observed an extensive contribution of BCs to the luminal lineage all along the ductal tree, indicating that multipotent BCs were present in all ductal regions during this early step of postnatal development (Fig. 3B-G). Next, we administered DOX from P10 to P15 and performed our analysis at P15 or after 2 weeks of chase (P29) (Fig. 3H). Although the initial labelling was uniform along the ductal tree, we only found signs of multipotent fate at the tips, as indicated by the presence of many YFP^+^ K8^+^ LCs (Fig. 3I-M). These results show that the progressive spatial restriction of basal multipotency to the distal tip and the lineage restriction of BCs along the duct occur as soon as P10. DOX administration from P16 to P21 led to the labelling of rare LCs after 2 weeks of chase (P35) (Fig. 3N-S), whereas BC tracing from P21 to P26 led only to BC labelling after 2 weeks chase (P40) suggesting that BCs were no longer differentiating to the luminal lineage (Fig. 3T-Y). The progressive switch from multipotency to unipotency was also observed in the other prostate lobes (data not shown). These data demonstrate that BCs undergo a switch from multipotency to unipotency relatively early during prostate postnatal development, at the onset of puberty, and that this lineage restriction occurs in a spatially graded manner with the tips being the multipotent niche during the mid-stage of postnatal development.

### Widespread distribution of multipotent and luminal-committed basal SCs at the early stage of postnatal development

**Fig. 4.**
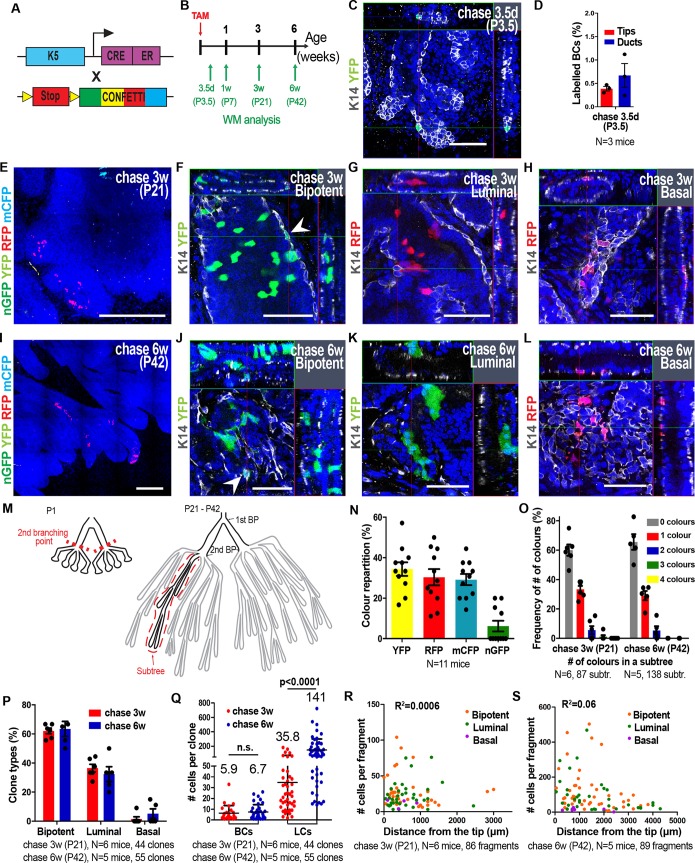
**Clonal analysis shows the widespread distribution of multipotent and luminal committed basal SCs at the early stage of postnatal prostate development.** (A) Genetic strategy used to label single and isolated K5-expressing BCs during early prostate postnatal development. (B) Temporal analysis of clonal lineage tracing. (C) Representative confocal image of recombined isolated BCs expressing one of the four colours of the Confetti reporter 3.5 days post-induction. (D) Quantification of labelled BCs at 3.5 days post-induction. (E) Confocal image of the VP induced at P1 and chased for 3 weeks. (F-H) Confocal images of clones derived from single labelled BCs 3 weeks post-induction. Arrowhead indicates a BC. (I) Confocal image of the VP induced at P1 and chased for 6 weeks. (J-L) Confocal images of clones derived from labelled BCs 6 weeks post-induction. Arrowhead indicates a BC. (M) Simplified drawing displaying the evolution of the ductal tree of the VP from P1 to P21-P42. The red dashed lines show the second branching point at P1 and outline a subtree at P21-P42 which is defined as the cluster of ducts arising after the second branching point of the prostate. BP, branching point. (N) Recombination frequency of the different colours of the Confetti reporter. (O) Frequency of observing the expression of the four Confetti colours within a ductal subtree. (P) Quantification of clone types (bipotent, luminal and basal unipotent) 3 and 6 weeks post-induction. (Q) Average basal and luminal clone sizes 3 and 6 weeks post-induction. (R,S) Distribution of the number of cells per clone relative to their distance from the tips 3 weeks (R) and 6 weeks (S) post-induction. In all image panels, K14 is visualised in grey and nuclei were counterstained with Hoechst 33342 in blue. Data show mean±s.e.m. (D) or mean±s.d. (N,O,P,Q). The number of clones quantified and the number of mice analysed are indicated in the respective panels. *P*-values were calculated by Mann–Whitney test. R^2^ was calculated from the Pearson correlation coefficient. n.s., not significant. Scale bars: 50 μm (C,F-H,J-L); 500 μm (E,I).

Our previous short-term clonal analysis of prostate BCs induced at P1 by imaging tissue sections suggested that either multiple types of multipotent and unipotent SCs co-exist at birth or that a single population of multipotent SCs divides asymmetrically giving rise to a multipotent SC and a unipotent luminal SC (Ousset et al., 2012). However, the absence of 3D information for the whole prostate and the fact that we performed the clonal analysis at a single time point (P1) prevented us from discriminating between these two possibilities. To investigate more precisely the heterogeneity of cell fate and the clonal dynamics of BCs during the early stage of postnatal development, we performed lineage tracing of BCs at clonal density at P1 and assessed their fate all along the ductal tree after 3 weeks and 6 weeks of chase using WM confocal microscopy. We used K5CreER^T2^ mice, because K5 is a cytokeratin expressed specifically in all BCs at the early stage of prostate development, and took advantage of the four-colour confetti construct to evaluate the clonality of our labelling strategy (Fig. 4A). We administered a low dose of tamoxifen (TAM) to P1 mice and scored the recombination frequency of the cells at different time points post-induction (Fig. 4B). We observed isolated BCs expressing one of the four confetti colours 3.5 days after labelling irrespective of their relative position within the ductal tree (Fig. 4C,D). The analysis of fluorescent patches by WM imaging 1, 3 and 6 weeks after induction demonstrated the presence of clusters of fluorescently labelled cells containing BCs and/or LCs along the ducts and the tip regions (Fig. 4E-L, Fig. S2A,D,E).

Soon after initial labelling (P7), fluorescence-marked clones formed small clusters of labelled cells that were cohesive (Fig. S2A). At later time points (P21, P42), these fluorescence-labelled cells were often not cohesive and were separated by non-labelled cells (Fig. 4E,I, Fig. S2D,E). To validate the clonality of our tracing experiments at P21 and P42, we scored the recombination frequency of each fluorescent protein (YFP, RFP, mCFP, nGFP) and the number of colours observed per ductal subtree. A subtree is defined as the different branches that share a common ancestor at the level of the second branch of a ductal tree, which exists prior to TAM administration (Fig. 4M-O). We found that 3 and 6 weeks after TAM administration, 60% of all subtrees were not labelled at all, the vast majority of labelled subtrees (>80%) were labelled with only one confetti colour and a minority of the ductal trees were labelled by more than one colour (Fig. 4O, Fig. S1F,G), indicating that the fluorescent patches of the same colour within a ductal tree are of clonal origin. Interestingly, clones of common origin were often separated by non-labelled cells (clone fragmentation) and appeared as non-continuous areas of labelled cells participating in the morphogenesis of several branches along the ductal tree, sometimes composed of different cell types depending on the branches (Fig. 4E,I).

To analyse the fate of BCs, we recorded the cellular composition of all clones and their location within the ductal tree 1, 3 and 6 weeks after induction. One week after labelling, 40% of clones were bipotent, 22% contained only LCs and 38% were composed only of BCs (Fig. S2B). At 3 and 6 weeks after induction, 60% of the clones were bipotent (containing BCs and LCs), 35% were composed exclusively of LCs and less than 5% contained only BCs (Fig. 4P). The relative proportion of bipotent and unipotent clones did not change between 3 and 6 weeks post-induction (Fig. 4P).

Next, we quantified the number of BCs and LCs in bipotent and unipotent clones. The number of BCs per clone was relatively homogeneous with no particularly big clones and the mean basal clone size was three cells at 1 week tracing, and six cells and seven cells at 3 and 6 weeks, respectively (Fig. 4Q, Fig. S2C,H,I). These data suggest that the basal compartment expanded by threefold from P1 to P7 and by twofold from P7 to P21, but it did not increase further from 3 to 6 weeks, consistent with the decrease in cell density of BCs found at the onset of puberty (Figs 4Q and 1O). The number of LCs per clone presented a much greater heterogeneity ranging from small clones containing few LCs to clones composed of more than 150 LCs at 3 weeks, up to 400-600 LCs at 6 weeks (Fig. 4Q, Fig. S2H,I). The mean number of LCs per clone was five cells at 1 week, 36 cells at 3 weeks and 141 cells at 6 weeks, showing the considerable and continuous expansion of the LC lineage during the whole process of prostate postnatal development until adulthood (Fig. 4Q, Fig. S2C).

To assess whether the clonal dynamic is influenced by its position along the ductal tree, we plotted the number of cells per fragment according to its position in relation to the end of the tip (0 μm). We found that fragment size did not have a strong correlation with its position along the duct (Fig. 4R,S), further suggesting that the SC activity drives the overall tissue expansion during early development.

Altogether, these data show that BCs consist of a heterogeneous population already at P1, with the majority of the BCs being multipotent and the rest committed to the LC lineage. Only a very small proportion of BCs remains purely basal during the course of prostate development.

### Multipotency is restricted to the tip region during mid postnatal prostate development

**Fig. 5.**
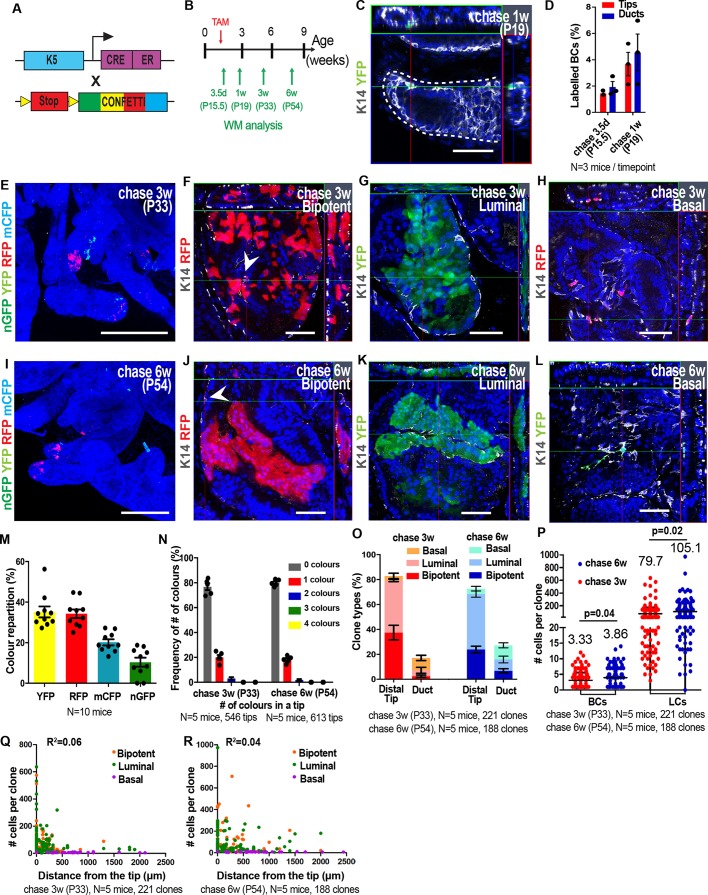
**Clonal analysis shows that multipotency is restricted to the tip region during mid postnatal prostate development.** (A) Genetic strategy used to label single and isolated K5-expressing BCs during mid prostate postnatal development. (B) Temporal analysis of clonal lineage tracing. (C) Confocal image of isolated recombined BCs expressing one of the four colours of the Confetti reporter 1 week post-induction. Dashed line highlights the epithelial structure. (D) Quantification of labelled BCs 3.5 and 7 days post-induction. (E) Confocal image of the VP induced at P12 and chased for 3 weeks. (F-H) Confocal images of clones derived from single labelled BCs 3 weeks post-induction. Arrowhead indicates a BC. (I) Confocal image of the VP induced at P12 and chased for 6 weeks. (J-L) Confocal images of clones derived from single BCs 6 weeks post-induction. Arrowhead indicates a BC. (M) Recombination frequency of the four colours of the Confetti reporter. (N) Frequency of observing the expression of the four Confetti colours in a tip. (O) Quantification of clone types (bipotent, luminal and basal unipotent) according to their spatial localisation 3 and 6 weeks post-induction, showing that almost all bipotent and luminal clones are found initially at the distal tip, and are progressively displaced more distally as ducts grow in length. (P) Average basal and luminal clone sizes 3 and 6 weeks post-induction. (Q,R) Distribution of the number of cells per clone relative to their position from the tips 3 weeks (Q) and 6 weeks (R) post-induction. In all image panels, K14 is visualised in grey and nuclei were counterstained with Hoechst 33342 in blue. Data show mean±s.e.m. (D) or mean±s.d. (M,N,O,P). The number of clones quantified and the number of mice analysed are indicated in the respective panels. *P*-values were calculated by Mann–Whitney test. R^2^ was calculated from the Pearson correlation coefficient. n.s., not significant. Scale bars: 50 μm (C,F-H,J-L); 500 μm (E,I).

**Fig. 6.**
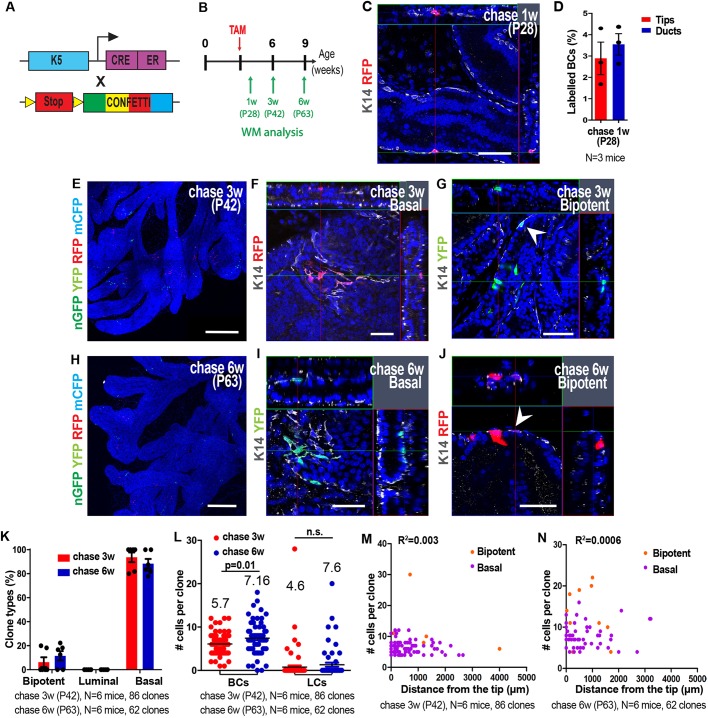
**Clonal analysis shows that basal stem cells become unipotent at the onset of puberty.** (A) Genetic strategy used to label single and isolated K5-expressing BCs during pubertal prostate development. (B) Temporal analysis of clonal lineage tracing. (C) Confocal image of isolated recombined BCs expressing one of the four colours of the Confetti reporter 1 week post-induction. (D) Quantification of labelled BCs 1 week post-induction. (E) Confocal image of the VP induced at P21 and chased for 3 weeks. (F,G) Confocal images of clones derived from single labelled BCs 3 weeks post-induction. Arrowhead indicates a BC. (H) Confocal image of the VP induced at P21 and chased for 6 weeks. (I,J) Confocal images of clones derived from single BCs 6 weeks post-induction. Arrowhead indicates a BC. (K) Quantification of clone types (bipotent, luminal and basal unipotent) 3 and 6 weeks post-induction, showing that 90% of basal cells are unipotent and less than 10% of the clones are multipotent after P21. (L) Average basal and luminal clone sizes 3 and 6 weeks post-induction. (M,N) Distribution of the number of cells per clone relative to their distance from the tips 3 weeks (M) and 6 weeks (N) post-induction. In all image panels, K14 is visualised in grey and nuclei were counterstained with Hoechst 33342 in blue. Data show mean±s.e.m. (D) or mean±s.d. (K,L). The number of clones quantified and the number of mice analysed are indicated in the respective panels. *P*-values were calculated by Mann–Whitney test. R^2^ was calculated from the Pearson correlation coefficient. n.s., not significant. Scale bars: 50 μm (C,F,G,I,J); 500 μm (E,H).

To verify whether the fate and the dynamics of BCs change during the course of postnatal prostate development, we performed clonal lineage tracing of BCs at later time points, at P12 (Fig. 5) and P21 (Fig. 6), and assessed the fate outcome of BCs 3 weeks and 6 weeks following TAM administration to K5CreER/Rosa-Confetti mice. Similarly to what we observed for the clonal tracing performed at P1, only isolated labelled BCs with no preference for the duct or the tip were observed at 3.5 days and 1 week after tracing at P12 (Fig. 5B-D). The analysis of WMs 3 and 6 weeks after induction showed the presence of clusters of fluorescently labelled cells containing BCs and/or LCs mainly at the tip regions (Fig. 5E-G,I-K, Fig. S3A,B). In addition, clusters containing only labelled BCs were observed (Fig. 5H,L). As in our P1 clonal tracing, the vast majority of tips were not fluorescently marked and most of the labelled tips contained only one colour, demonstrating the clonality of these lineage experiments (Fig. 5M,N, Fig. S2C,D).

The clone composition (percentage and number of BCs and LCs per clone) was determined by confocal microscopy of WMs 3 weeks and 6 weeks after induction. We scored the clone composition according to their relative position along the ductal tree. For these measurements, we segmented the tissue into two different regions including the distal part of the duct (‘Distal tip’, up to 500 μm), and the remaining duct (‘Duct’, greater than 600 μm from the tip). Most of the clones with bipotent basal and luminal fate or only luminal fate were found around the distal tip 3 and 6 weeks after BC labelling at P12, suggesting that multipotent BCs are localised exclusively close to the tip region at this stage of development whereas BCs along the duct are already lineage restricted (Fig. 5O). Moreover, the percentage of bipotent and luminal clones located along the ducts increased 6 weeks after induction (Fig. 5O), suggesting that the net growth of the duct arises also from the production of cells at the tip region, which progressively transfers the newly generated LCs to a more proximal region of the duct.

The average number of BCs per clone was very small (one to six BCs) in comparison with the average number of LCs per clone, which was approximately 80 LCs and 106 LCs 3 and 6 weeks after P12 induction, respectively (Fig. 5P, Fig. S3E,F). Interestingly, some clones contained more than 200 LCs. The clones with the higher number of BCs were unipotent with no LCs (Fig. S3E,F), demonstrating that unipotent and multipotent BCs could divide at the same rate, with unipotent BCs dividing symmetrically and multipotent BCs dividing mostly asymmetrically giving rise to a BC and a LC, leading to expansion of the pool of LC progenitors. The number of cells per clone increased with proximity to the tip region (Fig. 5Q,R), supporting the notion that multipotent SC activity around P12 is located around the tip region and is responsible for the net local expansion of the prostate epithelium.

### Basal stem cells become unipotent at the onset of puberty

Clonal tracing in K5CreER/Rosa-Confetti mice at P21 and analysis 1 week after induction revealed initial labelling of BCs along the duct at very low density (Fig. 6A-D). Analysis performed 3 weeks (P42) and 6 weeks (P63) after induction demonstrated that most BCs are unipotent regardless of their location along the duct or at the tip region (Fig. 6E-J,M,N, Fig. S3G,H). Moreover, less than 10% of all clones presented some LCs and the number of LCs per clone was limited to an average of five and eight cells 3 and 6 weeks after induction, respectively (Fig. 6K,L). These findings show that the important BC contribution to the luminal lineage ends after P21 and suggest that LC expansion, which accompanies puberty, is mediated essentially by unipotent luminal SCs that were specified earlier during development.

### Important contribution of unipotent luminal SCs to LC expansion during postnatal development

**Fig. 7.**
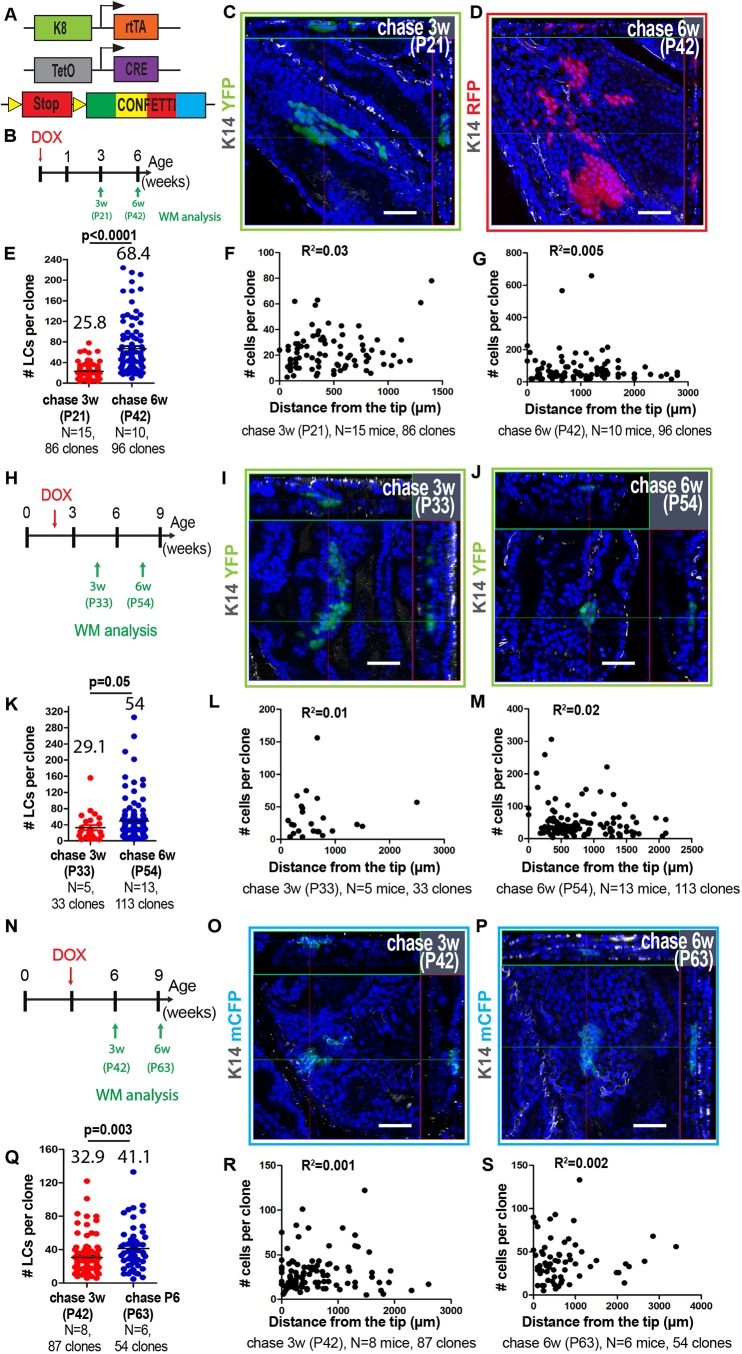
**Clonal analysis shows the important contribution of unipotent luminal stem cells to luminal lineage expansion during prostate development.** (A) Genetic strategy used to label K8-expressing LCs during prostate postnatal development. (B) Temporal analysis of clonal lineage tracing performed at P1. (C,D) Confocal images of clones derived from labelled LCs 3 weeks (C) and 6 weeks (D) post-induction. (E) Average luminal clone sizes 3 and 6 weeks post-induction. (F,G) Number of cells per clone relative to their distance to the tip region from 3 (F) and 6 (G) weeks post-induction at P1. (H) Temporal analysis of clonal lineage tracing performed at P12. (I,J) Confocal images of clones derived from labelled LCs 3 weeks (I) and 6 weeks (J) post-induction. (K) Average luminal clone sizes 3 and 6 weeks post-induction. (L,M) Number of cells per clone relative to their distance to the tip region 3 (F) and 6 (G) weeks post-induction. (N) Temporal analysis of clonal lineage tracing performed at the onset of puberty (P21). (O,P) Confocal images of clones derived from labelled LCs 3 weeks (O) and 6 weeks (P) post-induction. (Q) Average luminal clone sizes 3 and 6 weeks post-induction. (R,S) Number of cells per clone relative to their distance to the tip region 3 (F) and 6 (G) weeks post-induction. K14 is visualised in grey and nuclei were counterstained with Hoechst 33342 in blue. Data show mean±s.d. (E,K,Q). The number of clones quantified and the number of mice analysed are indicated in the respective panels. *P*-values were calculated by Mann–Whitney test. R^2^ was calculated from the Pearson correlation coefficient. Scale bars: 50 μm.

To determine directly the importance of unipotent luminal SCs in ensuring luminal lineage expansion during prostate postnatal development, we performed clonal analysis of luminal-targeted cells using K8rtTA/TetO-Cre/Rosa-Confetti mice at different time points during postnatal development (Fig. 7A). Administration of DOX to mice at P1 led to luminal clones of various sizes ranging from 10 to 50 LCs (26 LCs on average) 3 weeks after induction and from 20 to 120 LCs (68 LCs on average) 6 weeks after induction (Fig. 7B-E, Fig. S4A,B). Interestingly, the luminal clones were not bigger close to the tip region and clones were evenly distributed along the ductal tree (Fig. 7F,G), suggesting that, once specified, the different luminal progenitors contribute similarly to the net expansion of the LC lineage during postnatal growth.

DOX administration at later time points (P12 and P21) similarly led to the formation of luminal clones of various sizes located along the ductal tree 3 weeks (P33 and P42) and 6 weeks (P54 and P63) post-induction (Fig. 7H-S, Fig. S4C-F). Interestingly, the average size of the clones at 6 weeks decreased with the time of DOX administration (dropping from 68 to 41; Fig. 7E,K,Q), consistent with the morphometric analysis showing that most of the prostate postnatal expansion occurs before P42.

## DISCUSSION

In this study, we uncover the spatiotemporal regulation of proliferation dynamics and multipotency during prostate postnatal development.

Our whole-tissue imaging provides insightful information regarding prostate growth. We show that early postnatal development is accompanied by a linear increase both in tissue size (length and width) and complexity (branching morphogenesis). The onset of puberty coincides with faster growth rate and expansion of the luminal compartment, suggesting that the hormonal changes during this period are associated with the induction of a new mode of tissue morphogenesis that maximises the secretory capacity of the organ as it reaches sexual maturity.

Using different lineage-tracing approaches induced at different time points during postnatal development, we demonstrate that the majority of basal SCs are multipotent or committed to the luminal lineage during the first days of prostate postnatal development. At that stage of postnatal development, multipotent basal SCs are present all along the ductal trees, giving rise to luminal progenitors, which exhibit extensive self-renewal capacities, expansion and differentiation potential. At that time, BCs also comprise committed luminal SCs or progenitors that give rise only to LCs, without any trace of their previous BC fate. As prostate development proceeds, the main ductal regions lose their multipotent fate and multipotent BCs become located exclusively at the tip regions, promoting the prostate remodelling and branching that precedes prostate growth during puberty. Once the final pattern of the future adult prostate is established, with all the definitive branches and tips, the final prostate expansion, which is associated with puberty, is mediated mainly by unipotent basal and unipotent luminal SCs and progenitors. In the prostate, this switch from multipotency to unipotency occurs during childhood before puberty. In contrast, in mammary gland (MG) development, which presents more advanced branching at birth compared with the prostate, the switch from multipotency to unipotency of BCs occurs during embryonic development between E15.5 and E18.5 and all postnatal development is mediated by unipotent SCs (Wuidart et al., 2016, 2018; Lilja et al., 2018). Moreover, LCs of the MG comprise at least two distinct lineages. One lineage co-expresses oestrogen and progesterone receptors whereas the other lineage does not express these receptors. Sweat glands are also initially developed by different classes of multipotent and lineage-restricted cells and become unipotent around 4 weeks of development (Lu et al., 2012). Interestingly, these different studies show that whereas various glandular epithelia present many similarities in the lineage differentiation potential of basal SCs switching from multipotent to unipotent SCs during the course of development, the temporal clock regulating this process is not identical among the different epithelia. It would be interesting to assess the mechanisms that regulate the temporal clock, and whether the mechanisms that restrict or activate multipotency are common across different glandular epithelia.

Multipotency is also an important process during prostate tumorigenesis. When the most frequently mutated tumour suppressor gene, *Pten*, is deleted from BCs, they reactivate a multipotent programme and begin to generate LCs before progressing into invasive tumours (Choi et al., 2012; Wang et al., 2013). Similar reactivation of multipotency has also been observed during MG tumorigenesis following the expression of oncogenic *Pik3ca* in adult BCs or LCs (Van Keymeulen et al., 2015). The gene signature that accompanies the reactivation of multipotency in these cells following oncogene expression resembles the gene signature that marks the embryonic multipotent SCs (Wuidart et al., 2018; Lilja et al., 2018), supporting the notion that reactivation of an embryonic programme and multipotency is a hallmark of tumorigenesis in glandular epithelia.

More studies are needed to understand the reasons why multipotency is actively suppressed in adult animals in physiological conditions, and why and how this multilineage fate potential is unleashed and reactivated during tissue repair, transplantation and oncogenic activation. It would be interesting to understand the nature of the niche surrounding the tip region that regulates multipotency during the mid-stage of prostate development. Do tip cells secrete specific signals that promote multipotency of BCs? What are the roles of the stromal and immune cues in the regulation of multipotency?

In conclusion, we identify in this study the spatiotemporal regulation of prostate postnatal morphogenesis, the spatial organisation of multipotent SCs and the temporal switch from multipotency to unipotency. These results will be instrumental to unravelling the molecular mechanisms that regulate multipotency during prostate development, regenerative conditions and tumorigenesis.

## MATERIALS AND METHODS

### Mice

K14rtTA (Nguyen et al., 2006) mice were provided by Elaine Fuchs, TetO-Cre (Perl et al., 2002) were provided by Andreas Nagy and Rosa26-Confetti (Snippert et al., 2010) mice were provided by Hans Clevers. Rosa26-YFP (Srinivas et al., 2001) and CD1 mice were obtained from the Jackson Laboratory. The generation of K5CreER^T2^ mice (Van Keymeulen et al., 2011) and K8rtTA mice (Watson et al., 2015) was previously described. The experimental mice used in this study were male and of mixed background. All animals were housed under standard laboratory conditions in a certified animal facility receiving food and water *ad libitum*. All experiments were conducted in compliance with European guidelines and ethical protocols (under protocol numbers 546N and 673N) were approved by the local ethical committee for animal welfare (CEBEA). No statistical methods were used to predetermine sample size. The experiments were not randomised. The investigators were not blinded to allocation during experiments and outcome assessment.

### Induction of YFP expression

For tracing at saturation, K14rtTA(h);TetO-Cre(h);R26-YFP(h) mice were induced with a single dose of DOX (Sigma-Aldrich; diluted in PBS) administrated by intraperitoneal injection at P1 (20 μl of 10 mg/ml), P10, P16 or P21 (100 μl of 10 mg/ml) and maintained on treatment for 5 days by oral administration of DOX diluted in drinking water (2 mg/ml, AG Scientific) provided to them or to the mother. Mice were analysed at the end of DOX treatment (no chase) or 2 weeks later (chase 2w). To validate leakiness of the system and non-specific (not K14) labelling, P1 mice received a single dose of DOX by intraperitoneal injection (20 μl of 10 mg/ml) and were sacrificed 1 day later.

### Induction of Confetti expression

For clonal lineage tracing of basal cells, K5CreER^T2^(h);R26-Confetti(h) mice were induced with a single dose of TAM (Sigma-Aldrich; diluted in sunflower seed oil, Sigma-Aldrich) administrated by intraperitoneal injection at P1 (20 μl of 2.5 mg/ml), at P12 (100 μl of 2 mg/ml) or at P21 (100 μl of 10 mg/ml) and sacrificed 3.5 days, 1 week, 3 weeks or 6 weeks later. For clonal lineage tracing of luminal cells, K8rtTA(h);TetO-Cre(h);R26-Confetti(h) mice were induced with a single dose of DOX (diluted in PBS) administrated by intraperitoneal injection at P1 (20 μl of 1 mg/ml), at P12 (100 μl of 0.2 mg/ml) or at P21 (100 μl of 0.8 mg/ml) and sacrificed 3 weeks or 6 weeks later.

### Cell proliferation assay

To trace proliferating cells, mice at different age were injected intraperitoneally with 12.5 mg/kg EdU (Molecular Probes; diluted in PBS) every 12 h. Animals were sacrificed 12 or 24 h after the first injection.

### Whole-mount prostate processing and immunofluorescence staining

Samples processed at P1, P2, P3, P3.5 and P5 were obtained by dissection of the entire urogenital system followed by removal of the bladder, the testicles and the fat tissue. For samples processed at later time points, prostate tissue was microdissected under a stereoscope to separate the different lobes. The ventral lobes were enzymatically digested in HBSS (Gibco) supplemented with 10 mg/ml collagenase (Sigma-Aldrich) for 2 to 8 min at room temperature (RT) depending on tissue size and animal age. Samples were rinsed twice with PBS for 5 min and fixed in 4% paraformaldehyde (PFA) for 2 h at RT. After two washes with PBS for 5 min, tissues were incubated in blocking buffer [1% bovine serum albumin (BSA), 5% horse serum (HS), 0.8% Triton X-100 in PBS] for 3 h at RT while shaking. Primary antibodies were diluted in blocking buffer and incubated overnight at RT while shaking. Lobes were washed three times (10 min each) with 0.2% Tween 20 in PBS, before being incubated for 4-5 h at RT on a rocking plate with the appropriate secondary antibody diluted in blocking buffer. Anti-GFP (chicken, 1:1000, ab13970, Abcam), anti-K14 (rabbit, 1:2000, PRB-155P-0100, Covance), anti-K5 (chicken, 1:2000, 905901, Covance) and anti-K8 TROMA-1 (rat, 1:800, Developmental Studies Hybridoma Bank) were used as primary antibodies. Anti-chicken, anti-rabbit and anti-rat conjugated to Alexa Fluor 488 (1:400, Molecular Probes, A11039, A21206, A21208), anti-rabbit conjugated to Rhodamine Red-X or to Cy5 (1:400, Jackson ImmunoResearch, 711-295-152, 711-605-152) and anti-chicken and anti-rat conjugated to Cy5 (1:400, Jackson ImmunoResearch, 703-605-155, 712-605-153) were used as secondary antibodies. To visualise proliferating cells that incorporated EdU, the Click-iT EdU Alexa Fluor 594 Imaging Kit (Molecular Probes) was used and the Click-iT reaction was performed according to manufacturer's instructions but with extended permabilisation to 0.8% Triton X-100 for 30 min followed by 1.2% Triton X-100 for 10 min. Subsequently, incubation to the EdU cocktail was extended to 40 min. Nuclei were counterstained using Hoechst 33342 dye (Sigma-Aldrich) (1:10,000 for Confetti mice or 1:1000 for YFP or EdU mice in PBS 0.2% Tween 20) for 30 min (Confetti or YFP mice) or 2 h (EdU mice) at RT while shaking. Tissues were washed twice in 0.2% Tween 20 in PBS for 10 min and mounted on a 1.5 mm coverslip (VWR) in glycergel mounting medium (DAKO) supplemented with 2.5% Dabco (Sigma-Aldrich).

### Histology and immunostaining on sections

Prostate tissue of mice at P21, P42 and P56 was microdissected under a stereoscope to separate the different lobes. Dissected prostates were pre-fixed in 4% PFA for 2 h at RT. After three washes with PBS for 5 min each wash, samples were incubated overnight in 30% sucrose in PBS at 4°C. Tissues were embedded in OCT compound (Tissue-Tek) and kept at −80°C. Sections of 5 μm thickness were cut using a HM560 Microm cryostat (Mikron Instrument). Sections were incubated in blocking buffer (1% BSA, 5% HS, 0.2% Triton X-100 in PBS) for 1 h at RT and then with primary antibodies overnight at 4°C. Tissues were rinsed three times in PBS, 5 min each, and incubated with secondary antibodies diluted in blocking buffer for 1 h at RT. Anti-K14 (chicken, 1:1000, PRB-155P-0100, Covance), anti-K8 TROMA-1 (rat, 1:1000, Developmental Studies Hybridoma Bank) and anti-cleaved caspase3 (Asp175) (rabbit 1:400, 9664S, Cell Signaling) were used as primary antibodies. Anti-rat conjugated to Alexa Fluor 488 (1:400, Molecular Probes, A21208), anti-chicken conjugated to Rhodamine Red-X (1:400, Jackson ImmunoResearch, 703-295-155) and anti-rabbit conjugated to Cy5 (1:400, Jackson ImmunoResearch, 711-605-152) were used as secondary antibodies. Nuclei were stained with Hoechst 33342 dye (Sigma-Aldrich) (diluted 1:1000 with the secondary antibodies) and slides were mounted in glycergel mounting medium (DAKO) supplemented with 2.5% Dabco (Sigma-Aldrich).

### Image acquisition and analysis

Confocal images were acquired at RT using a Zeiss LSM780 confocal microscope fitted on a Axiovert M200 inverted microscope equipped with a LD C Apochromat (40×, NA=1.1) water immersion objective (Carl Zeiss). Optical sections (512×512 pixels) were collected sequentially for each fluorochrome. Images of maps reconstructing the whole mounts were obtained at RT using a LD LCI Plan Apochromat (25×, NA=0.8) water & glycerol immersion objective (Carl Zeiss) or a Plan Apochromat (20×, NA=0.8) without immersion objective (Carl Zeiss). Optical sections (512×512 or 256×256 pixels, depending on map size) were collected sequentially for each fluorochrome. The generated data were processed and displayed using ZEN software. Maps were cropped if necessary to isolate the prostate epithelium from mesenchyme and surrounding tissue. Quantifications were performed manually using the scoring tool of ZEN software.

### Clonal analysis

Clone size and composition was checked and scored manually. To assess clonality in K5CreER^T2^(h);R26-Confetti(h) mice induced at P1 after 3 and 6 weeks of tracing, the number of Confetti colours per subtree and the colour repartition were scored. Using the frequency of observing 0 colours per subtree, the probability of observing 1, 2, 3 and 4 colours per subtree was estimated from the Poissonian distribution. To asses clonality in K5CreER^T2^(h);R26-Confetti(h) mice induced at P12 after 3 and 6 weeks of tracing, the number of Confetti colours per tip and the colour repartition were scored. Using the frequency of observing 0 colours per tip, the probability of observing 1, 2, 3 and 4 colours per tip was estimated from the Poissonian distribution. To assess clonality in K5CreER^T2^(h);R26-Confetti(h) mice induced at P21, maps were macroscopically examined and no marked cells were found within a distance of 500 μm from the clones of marked cells. To assess clonality in K8rtTA(h);TetO-Cre(h);R26-Confetti(h) mice induced at P1, P12 or P21 after 3 and 6 weeks of tracing, only clones that were isolated at least 500 μm from other labelled cells were scored.

### Statistics

Statistical and graphical data analyses were performed using Prism 6 (GraphPad) software. All data in histograms represent mean±s.e.m. or ±s.d. Data were tested for normality using the D'Agostino and Pearson omnibus normality test. Statistical significance was calculated by Mann–Whitney, considering *P*<0.05 as statistically significant. R^2^ was calculated by computing the Pearson correlation coefficient. All tests were two-sided.

## Supplementary Material

Supplementary information
