# Supplementary material for: Spatiotemporal regulation of multipotency during prostate development
Source: Development. 2019 Oct 23;146(20):dev180224. doi: 10.1242/dev.180224 (PMC6883376; doi:10.1242/dev.180224)
Supplement: Supplementary information [file develop-146-180224-s1.pdf]

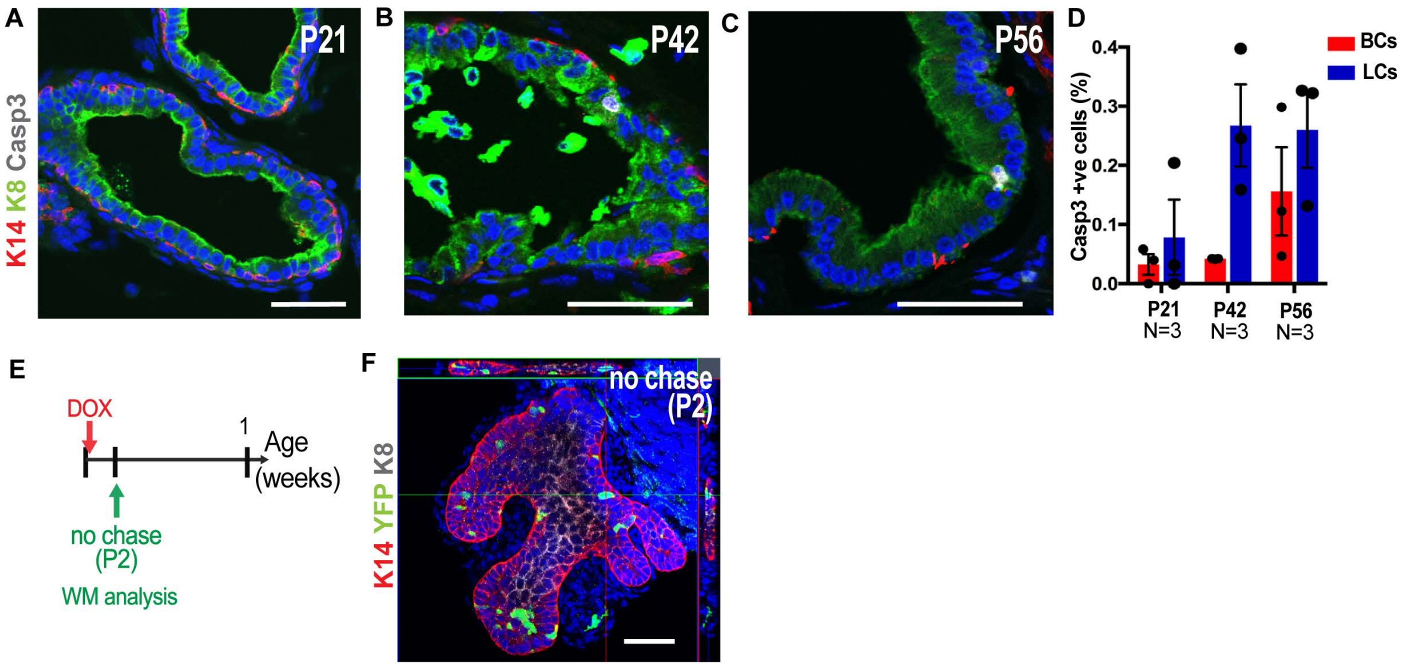

Fig. S1 (Related to Fig. 2 and Fig. 3) Analysis of Cell death and lineage tracing specificity during prostate development.

(A-C) Representative images of immunostaining of Caspase3 (grey), K14 (red) and K8 (green) on prostate sections at mice of age P21 (A), P42 (B) and P56 (C). (D) Quantification of Caspase3 positive cells at different time points during prostate development. Mean±SEM are shown from 14,016, 53,732 and 49,410 cells counted at the different time points indicated (left to right). (E) Protocol used to analyse the initial targeted cells of saturation lineage tracing at early stage of postnatal development (P1-P2). (F) Confocal image of the VP at the end of Dox administration (P2). K14 is visualized in red, YFP in green, K8 in grey. In all image panels, nuclei were counterstained with Hoechst 33342 in blue. Scale bar, 50μm.The number of mice analysed are indicated in the respective panels.

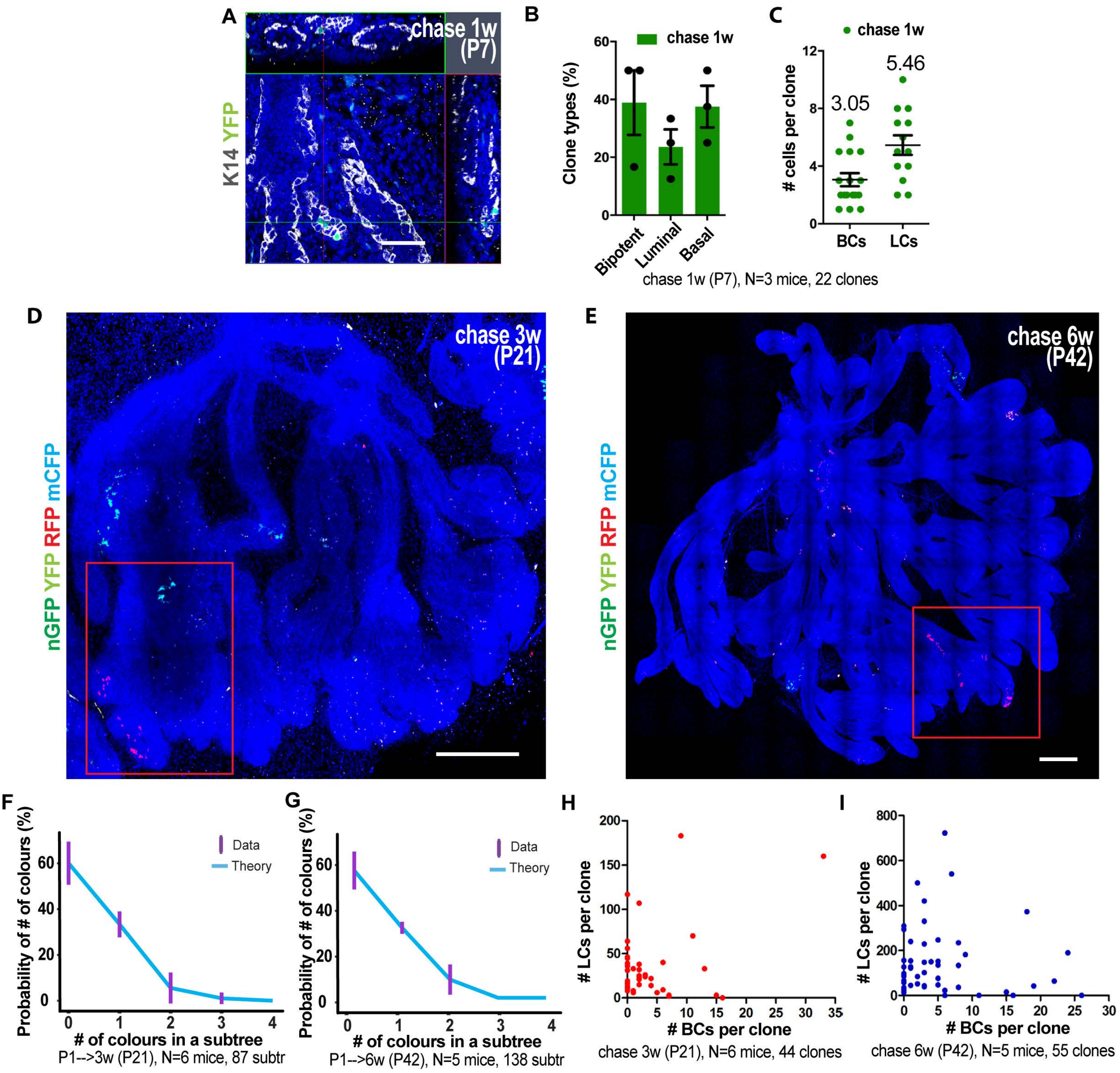

Fig. S2 (Related to Fig. 4) The contribution of multipotent basal progenitors to the prostate postnatal development analysed by confocal imaging of whole mount tissue.

(A) Representative confocal image of a clone derived from a single labeled BCs 1 week post induction. (B) Quantification of clone types (bipotent, luminal and basal unipotent) 1 week post induction. (C) Average basal and luminal clone sizes 1 week post induction.(D,E) Confocal images of the VP induced at P1 and chased for 3 weeks (D) or 6 weeks (E). Red panels are shown in higher magnification in Fig. 4E and I. (F,G) Theoretical Poisson distribution of the number of colours obtained after clonal marking in a subtree at 3 (F) and 6 (G) weeks post induction. (H,I) Number of BCs and LCs per clone derived from labeling of single BCs 3 weeks (H) and 6 weeks (I) post induction. In all image panels K14 is visualized in grey and nuclei were counterstained with Hoechst 33342 in blue. Data show mean±SD (B,C). Scale bar, 50μm (A) and 500μm (D,E).The number of subtrees and clones quantified and the number of mice analysed are indicated in the respective panels.

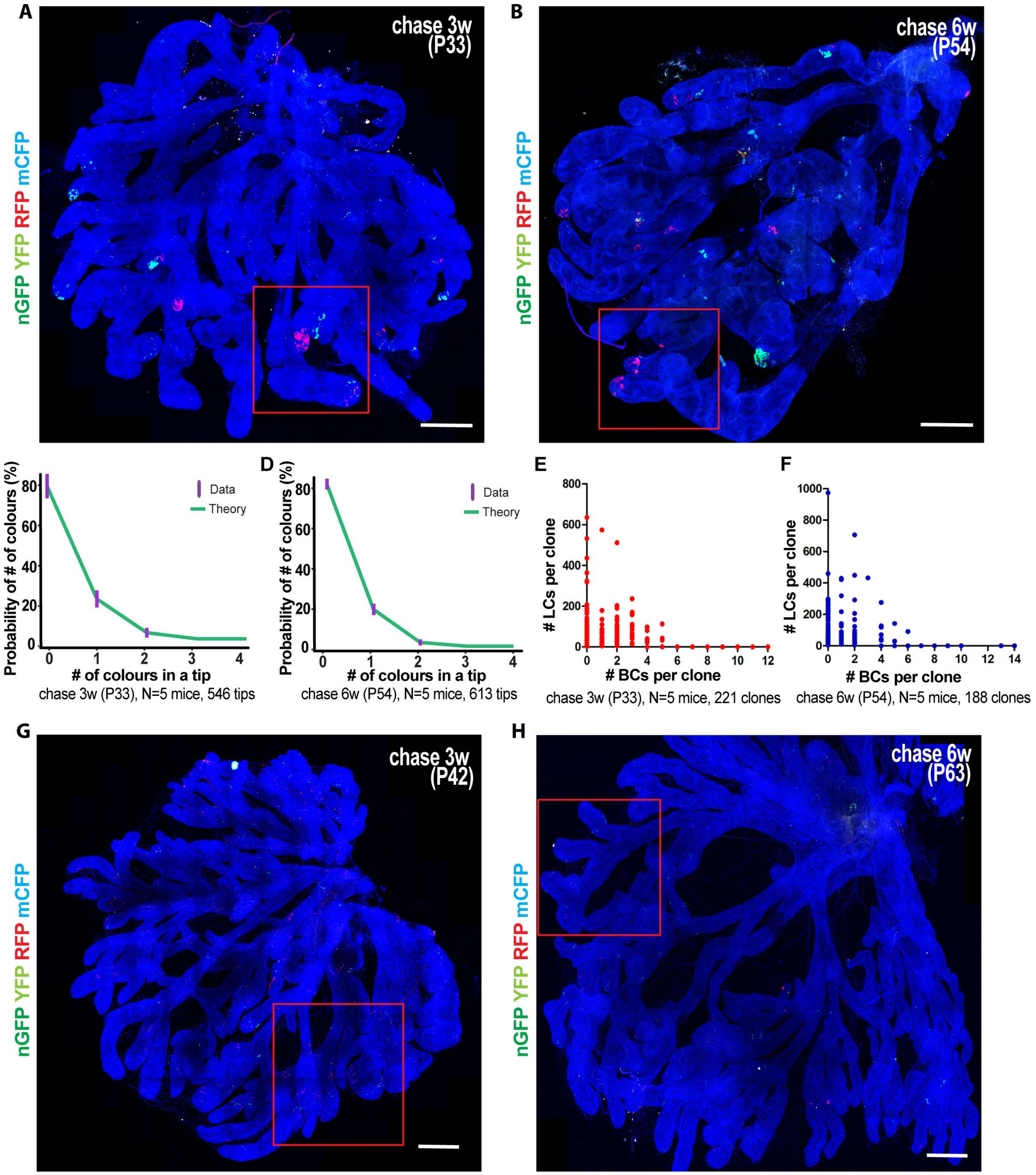

Fig. S3 (Related to Fig. 5 and Fig. 6) The spatiotemporal restriction of multipotency during prostate development.

(A,B) Confocal images of the VP induced at P12 and chase for 3 weeks (A) or 6 weeks (B). Red panels are shown in higher magnification in Fig. 5E and I. (C,D) Theoretical Poisson distribution of the number of colours obtained after clonal marking in a tip 3 (C) and 6 (D) weeks post induction. (E,F) Number of BCs and LCs per clone derived from labeling of single BCs 3 weeks (E) and 6 weeks (F) post induction. (G,H) Confocal images of the VP induced at P21 and chased for 3 weeks (G) or 6 weeks (H). Red panels are shown in higher magnification in Fig. 6E and H. In all image panels nuclei were counterstained with Hoechst 33342 in blue. Scale bar, 500μm. The number of tips and clones quantified and the number of mice analysed are indicated in the respective panels.

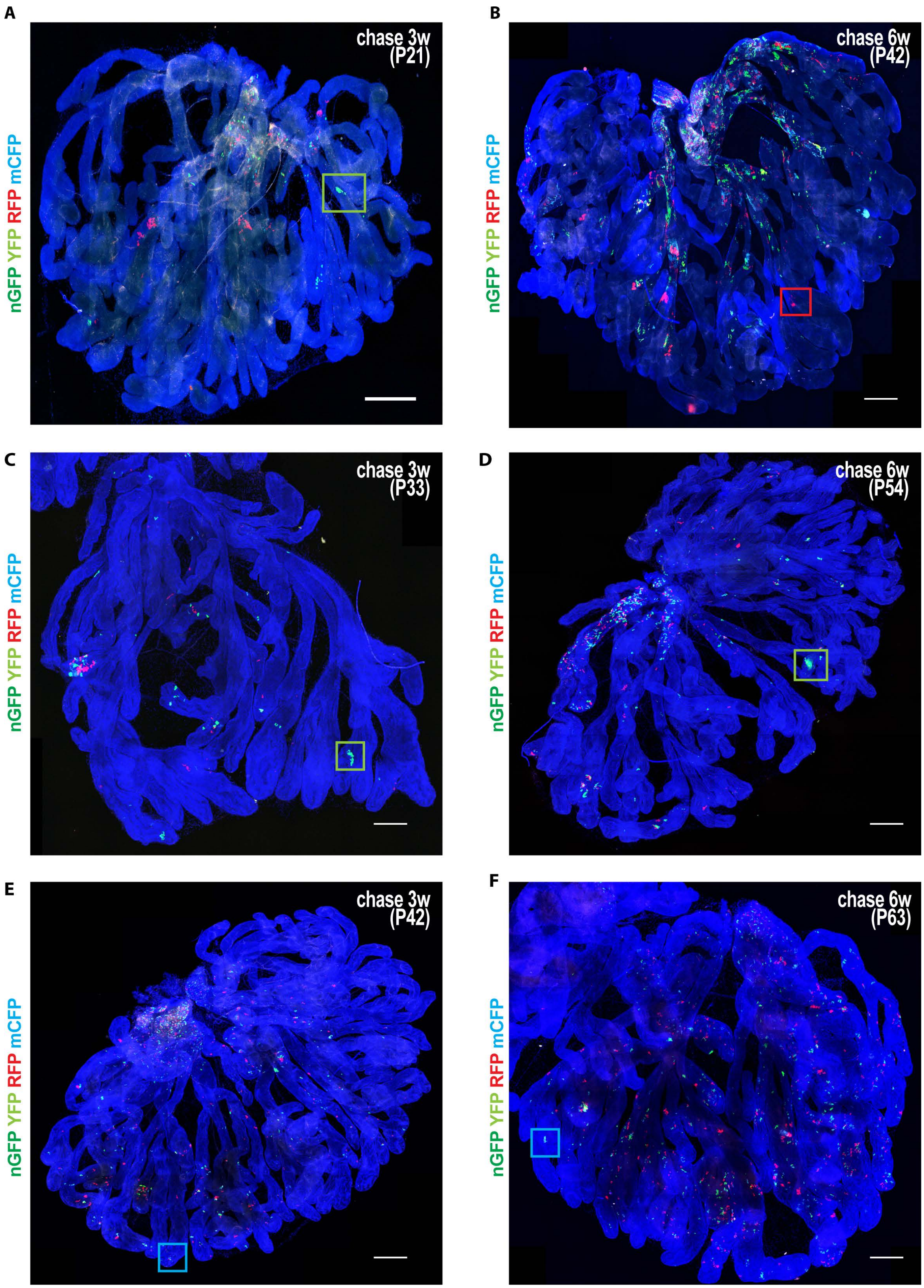

Development • Supplementary information

Fig. S4 (Related to Fig. 7) Confocal analysis of the fate of unipotent luminal stem and progenitor cells during prostate development.
(A,B) Confocal images of the VP induced at P1 and chase for 3 weeks (A) or 6 weeks (B). (C,D) Confocal images of the VP at P12 and chased for 3 weeks (C) or 6 weeks (D). (E,F) Confocal images of the VP induced at P21 and chased for 3 weeks (E) or 6 weeks (F). In all image panels, rectangles are clones represented in higher magnification in Fig. 7, nuclei were counterstained with Hoechst 33342 in blue. Scale bar, 500µm.
